# Childhood executive functions and ADHD symptoms predict psychopathology symptoms in emerging adults with and without ADHD: a 10-year longitudinal study

**DOI:** 10.1007/s10802-022-00957-7

**Published:** 2022-10-04

**Authors:** Stian Orm, Per Normann Andersen, Martin Hersch Teicher, Ingrid Nesdal Fossum, Merete Glenne Øie, Erik Winther Skogli

**Affiliations:** 1 Division Mental Health Care, Innlandet Hospital Trust, BUP Lillehammer, Anders Sandvigs gate 17, 2629 Lillehammer, Norway; 2 Department of Psychology, University of Oslo, Oslo, Norway; 3 Department of Psychology, Inland Norway University of Applied Sciences, Hamar, Norway; 4 Harvard Medical School, Boston, United States; 5 Research Department, Innlandet Hospital Trust, Brumunddal, Norway

**Keywords:** Attention-Deficit/Hyperactivity disorder, Executive functions, Cognitive flexibility, Psychopathology, Longitudinal, Comorbidity

## Abstract

Deficits in executive functions (EFs) are theorized to play an important role in causing functional impairment and associated psychopathology in individuals with ADHD. The objective of this study was to examine the role of EFs and ADHD symptoms as longitudinal predictors of psychopathology symptoms in individuals with ADHD and typically developing individuals. We assessed individuals with and without ADHD (*N* = 135) with neuropsychological tests of EFs and scales of ADHD symptoms and psychopathology symptoms at baseline (T1; M_age_ = 11.59, 57.8% boys), 2-year follow-up (T2; M_age_ = 13.63, 97% retention), and 10-year follow-up (M_age_ = 21.18, 75% retention). Baseline EFs predicted psychopathology symptoms at the 2- and the 10-year follow-up, explaining 17% and 12% of the variance, respectively. Baseline EFs predicted both internalizing and externalizing symptoms, and the predictive value of EFs on psychopathology symptoms at 10-year follow-up was accounted for by cognitive flexibility. Baseline ADHD symptoms were a significant predictor of all symptom domains at all time points. Thus, childhood EFs, in particular cognitive flexibility, can predict psychopathology symptoms in emerging adulthood beyond the effect of ADHD symptoms. This supports dominating theories of ADHD stating that executive dysfunction contributes to the observed phenotype, including associated psychopathology symptoms, and suggests that EFs are important targets of interventional efforts.

Numerous studies have found that many individuals with Attention-Deficit/Hyperactivity Disorder (ADHD), even those without a comorbid disorder, display heightened levels of internalizing and externalizing symptoms compared with typically developing (TD) individuals (e.g., Butzbach et al., 2021; Skogli et al., 2013). These psychopathology symptoms can be troublesome and impact functional domains such as academic performances, quality of life, and occupational and family functioning (Schei et al., 2016; Sobanski et al., 2007; Zendarski et al., 2021). Despite the importance of these co-occurring psychopathology symptoms, little is known about which factors predict psychopathology symptoms. The current study uses data from a 10-year longitudinal study to address this research gap. We focus on the role of executive functions (EFs), as these abilities are central to understanding the nature, causes, and consequences of ADHD (Barkley, 1997; Fossum et al., 2021).

EFs are an umbrella term for effortful, top-down cognitive processes necessary for the regulation of other cognitive processes, behavior, and emotions, and initiation of goal-directed behavior (Diamond, 2013; Miyake et al., 2000). Three core EFs are response inhibition, working memory, and cognitive flexibility (Diamond, 2013; Miyake et al., 2000). Longitudinal studies show that ADHD is associated with long-term impairments in EFs from childhood and into adulthood (Fossum et al., 2021; Gordon & Hinshaw, 2020; Øie et al., 2021), with executive dysfunction contributing to poorer academic performance, occupational functioning, and quality of life (Barkley & Fischer, 2011; Stern et al., 2017; Tamm et al., 2021).

Some studies have also found that childhood EFs are predictive of later psychopathology symptoms in adolescents and emerging adults with ADHD (Meza et al., 2020; Miller et al., 2012; Owens & Hinshaw, 2016). Rinsky & Hinshaw (2011) found that poorer EFs predicted more psychopathology symptoms five years later, in adolescence. Miller et al., (2012) and Meza et al., (2020) found that poorer childhood EFs predicted a higher number of non-suicidal self-injury episodes and suicide attempts in emerging adulthood. Owens & Hinshaw (2016) examined the relationship between childhood EFs and psychopathology symptoms in emerging adulthood and possible mediators of this relationship. The authors found that poorer childhood EFs predicted more emerging adulthood psychopathology symptoms through two pathways; (1) through poorer early-adolescent academic performance and late-adolescent school failure, and (2) through poorer adolescent self-control.

These studies examining the predictive value of EFs on psychopathology symptoms have some limitations. First, all of these studies were based on the same sample of participants, the Berkeley Girls Longitudinal Study (BGLS; Owens et al., 2017). Thus, replication in other samples is warranted. Second, the Berkeley Girls sample consists exclusively of girls, meaning that it is uncertain whether the results can be generalized to the whole ADHD population. In a recent four-year longitudinal study of a mixed-sex sample (67% male) of children with ADHD, no predictive value of EFs was found on later depressive symptoms (Fenesy & Lee, 2022). Third, all of the studies from the BGLS used the same tests of EFs, and thus, the predictive value of other tests of EFs is unknown. Fourth, these studies examined the impact of each test of EFs separately. Thus, the combined predictive value of EFs on co-occurring symptoms and the extent to which EFs predict psychopathology symptoms in conjunction with other factors such as ADHD symptoms remains unclear. There is an ongoing debate on whether EFs are a unitary or multifactorial construct, with some evidence suggesting that a global EFs composite may be a better indicator of an individual’s EFs and have better predictive value than more circumscribed and specific measures (Øie et al., 2021; Owens & Hinshaw, 2016; Skogli et al., 2020). This may especially apply to children and adolescents, where a systematic review concluded that there is greater support for unidimensional rather than multifactorial models (Karr et al., 2018).

The current study aimed to investigate the ability of EFs and ADHD symptoms to predict psychopathology symptoms from a concurrent and longitudinal perspective. ADHD symptoms were included because ADHD symptoms have been found to associate with psychopathology symptoms across the general population and among individuals with ADHD, and thus can explain variation in psychopathology symptoms beyond group status (Cadman et al., 2016; Fenesy & Lee, 2022; Stern et al., 2020). Based on the current literature, we hypothesized that greater deficits in EFs and more ADHD symptoms would be associated with more psychopathology symptoms at baseline (T1) and predict psychopathology symptoms longitudinally at the 2- (T2) and 10-year (T3) follow-up assessments. In line with recent advances in the understanding of psychopathology (i.e., the *p-*factor, see Caspi & Moffitt 2018), we focused on a composite measure of total psychopathology symptoms, but we also explored the impact of EFs on the two narrower domains of internalizing (e.g., anxiety, depressive symptoms) and externalizing (e.g., conduct, aggressive symptoms) symptoms. Given that previous studies finding a predictive value of EFs on later psychopathology symptoms have been conducted exclusively with females, we also explored the possible moderating role of sex on the relationship between EFs and psychopathology symptoms. Lastly, since a recent study (Romer & Pizzagalli, 2021) suggested that the prospective relationship between EFs and psychopathology symptoms may be bidirectional, we also explored whether childhood psychopathology symptoms predict later EFs.

## Method

### Procedure

The study was prospectively reviewed and approved by the Regional Committee for Medical Research Ethics in Eastern Norway (T1-T2: REK 6-2009-24, T3: 2018/1611). Children between 8 and 17 years of age, who were consecutively referred to a child psychiatric clinic for assessment of ADHD, were invited to participate if they met the DSM-IV criteria for ADHD. The diagnostic assessment included the Kiddie-Schedule for Affective Disorders and Schizophrenia/Present and lifetime version (Kiddie-SADS; Kaufman et al., 1997), parent report on the ADHD Rating Scale-IV (ARS-IV; DuPaul et al., 1998a, b), and teacher reports on academic and social functioning (see Skogli et al., (2013) for further details). TD individuals were recruited from local schools and were screened for psychopathology with Kiddie-SADS. Exclusion criteria for all participants were prematurity (< 36 weeks), having a disease affecting the central nervous system, or having an IQ < 70. An additional exclusion criterion for the ADHD group was a history of stimulant treatment. Additional exclusion criteria for the TD group were a history of psychiatric disorder, dyslexia, or head injury with loss of consciousness.

## Participants and Sample Retention

At T1, a total of 135 children participated, 85 individuals with ADHD and 50 TD individuals. Demographic and clinical characteristics are presented in Table 1. Due to the high level of comorbidity among individuals with ADHD, participants with comorbid disorders were not excluded. Nine participants had comorbid depressive or anxiety disorder, two had conduct disorder, and nine had an oppositional defiant disorder. Unfortunately, no data was collected on the participants ethnicity.

**Table 1:** Demographic and clinical characteristics of participants at baseline, 2- and 10-year follow-up with group comparisons

Baseline (T1)	ADHD *n* = 85		TD *n* = 50		Group comparison
	*M*	*SD*	*M*	*SD*	*p*
Sex (% male/female)	54/46		64/36		0.262
Age (years)	11.6	2.1	11.6	2.0	0.887
FSIQ	94.4	13.8	103.8	13.0	< 0.001
Mother’s education (years)	12.7	2.2	14.6	2.4	< 0.001
ARS-IV	26.6	10.5	2.6	3.0	< 0.001
CBCL Total problems	61.9	8.2	37.9	8.7	< 0.001
CBCL Internalizing	59.3	10.5	42.4	8.7	< 0.001
CBCL Externalizing	59.9	10.5	40.8	7.5	< 0.001
2-year follow-up (T2)	ADHD *n* = 81		TD *n* = 50		
	*M*	*SD*	*M*	*SD*	*p*
Sample retention (%)	95.3		100.0		0.119
Sex (% male/female)	53/47		64/36		0.220
Age (years)	13.6	2.1	13.6	2.0	0.953
ARS-IV	18.2	10.9	2.2	2.7	< 0.001
CBCL Total problems	58.0	9.7	36.6	8.4	< 0.001
CBCL Internalizing	56.6	11.6	41.1	8.3	< 0.001
CBCL Externalizing	55.4	10.3	39.7	5.6	< 0.001
10-year follow-up (T3)	ADHD *n* = 61		TD *n* = 40		
	*M*	*SD*	*M*	*SD*	*p*
Sample retention (%)	71.8		80.0		0.287
Sex (% male/female)	56/44		65/35		0.354
Age (years)	21.4	2.3	20.9	1.9	0.228
ASR Total problems	54.7	12.5	44.0	10.3	< 0.001
ASR Internalizing	53.8	12.6	45.9	10.4	0.001
ASR Externalizing	51.5	11.4	43.3	8.8	< 0.001

*Note.* ADHD = attention-deficit/hyperactivity disorder; TD = typically developing; FSIQ = Full Scale IQ, estimated from Wechsler Abbreviated Scale of Intelligence; ARS-IV = ADHD Rating Scale IV; CBCL = Child Behavior Checklist; ASR = Adult Self-Report

At T2, 131 (97%) of the original 135 participants were retained. At T3, 101 (75%) of the original 135 participants were retained (see Table 2). We analyzed differences between retained participants and non-retained participants in T1 demographic characteristics (i.e., full-scale IQ [FSIQ], age, sex, mothers’ educational level) and the variables included in the current study (i.e., psychopathology symptoms; EFs; ADHD symptoms). There was evidence of selective attrition due to lower FSIQ, poorer working memory, and poorer cognitive flexibility. No significant differences were found between those retained and those not retained on ADHD symptoms or psychopathology symptoms. At T2 and T3, 54% and 12%, respectively, of individuals in the ADHD group received stimulant treatment.

**Table 2:** Differences in demographic and clinical characteristics between participants retained at 10-year follow-up (T3) and participants not retained

Baseline (T1)	Retained *n* = 101		Non-retained *n* = 34		Group comparison
	*M*	*SD*	*M*	*SD*	*p*
Sex (% male/female)	59/41		53/47		0.509
Age (years)	11.6	2.0	11.6	2.1	0.912
FSIQ	99.4	13.9	93.6	14.2	0.040
Mother’s education (years)	13.4	2.4	13.2	2.4	0.707
ARS-IV	16.2	14.0	19.6	14.1	0.217
CBCL Total problems	52.4	14.7	55.3	13.3	0.315
CBCL Internalizing	52.5	12.9	54.9	12.4	0.352
CBCL Externalizing	52.7	13.3	53.5	13.2	0.739
Letter-Number Sequencing Test	16.9	2.9	15.4	3.4	0.013
Color-Word Interference Test, Condition 3	79.9	27.6	88.2	29.3	0.136
Color-Word Interference Test, Condition 4	86.4	23.7	98.5	32.5	0.052
Trail Making Test, Condition 4	111.7	44.1	126.0	50.9	0.127

*Note.* FSIQ = Full Scale IQ estimated from Wechsler Abbreviated Scale of Intelligence, ARS-IV = ADHD Rating Scale IV edition, CBCL = Child Behavior Checklist

Table 3

**Table 3:** Results on the neuropsychological tests of executive functions and the composite score by group and across time

Variable	ADHD			TD		
	T1	T2	T3	T1	T2	T3
Letter-Number Sequencing Test ^a^	15.5 (3.3)	17.1 (2.9)	17.9 (2.9)	18.2 (1.9)	19.3 (2.4)	21.2 (2.7)
Color-Word Interference Test, Condition 3 ^b^	87.5 (29.8)	72.0 (27.3)	53.7 (15.8)	72.5 (22.3)	58.7 (16.9)	41.9 (9.0)
Color-Word Interference Test, Condition 4 ^b^	96.5 (28.0)	77.3 (23.8)	61.1 (14.5)	77.5 (19.0)	64.2 (14.7)	50.0 (9.9)
Trail Making Test, Condition 4 ^c^	124.4 (49.5)	99.9 (41.4)	74.0 (26.9)	99.9 (35.2)	76.5 (23.4)	62.3 (22.3)
Executive function composite score ^d^	2.9 (4.2)	0.2 (3.7)	-2.7 (3.0)	0.0 (3.1)	-2.5 (2.7)	-5.8 (3.0)

*Note.* ASD = autism spectrum disorder; ADHD = attention-deficit/hyperactivity disorder; TD = typically developing; T1 = baseline; T2 = 2-year follow-up; T3 = 10-year follow-up

^a^ raw score, higher scores = better performance. From Wechsler Intelligence Scales for Children-IV. T1: ADHD *n* = 83, TD *n* = 50. T2: ADHD *n* = 75, TD *n* = 50. T3: ADHD *n* = 60, TD *n* = 40

^b^ completion time in seconds, lower scores = better performance. From Delis-Kaplan Executive Function System (D-KEFS). T1: ADHD *n* = 85, TD *n* = 50. T2: ADHD *n* = 78, TD *n* = 50. T3: ADHD *n* = 60, TD *n* = 40

^c^ completion time in seconds, lower scores = better performance. From D-KEFS. T1: ADHD *n* = 82, TD *n* = 50. T2: ADHD *n* = 77, TD *n* = 50. T3: ADHD *n* = 60, TD *n* = 39

^d^ lower scores = better performance

## Measures

### Measures of psychopathology symptoms

We used the Achenbach System of Empirically Based Assessment (ASEBA; Achenbach & Rescorla 2001, 2003) to measure psychopathology symptoms. At T1 and T2, we used the parent-reported Child Behavior Checklist (CBCL; Achenbach & Rescorla 2001) and at T3, we used the Adult Self-Report (ASR; Achenbach & Rescorla 2003). We used the total problems composite score which comprises all subscales, including the two broader band composite scores of internalizing and externalizing behaviors. Raw scores were converted to age and sex-adjusted T-scores (M = 50, SD = 10). Psychometrically, the CBCL and ASR have good levels of reliability (α ≥ 0.80), sensitivity (40–83%), and specificity (70–94%) in general population samples (e.g., de Vries et al., 2020; Nøvik, 1999, 2000).

### Measures of executive functions

We used the total correct recalled trials score on the Letter-Number Sequencing Test (LNS; Wechsler 2004) as a measure of working memory (see Table 3). In this test, the test administrator reads aloud a sequence of letters and numbers and asks the participant to recall the letters in alphabetical order and the numbers in ascending order. Higher scores indicate better performance. The test is widely used and has demonstrated good psychometric properties (mean reliability = 0.75) in the general population and clinical populations (Baron, 2005; San Miguel Montes et al., 2010).

We used the completion time on the Color-Word Interference Test, condition 3 (CW3; Delis et al., 2001) as an index of response inhibition and completion time on the Color-Word Interference Test, condition 4 (CW4; Delis et al., 2001) as a measure of cognitive flexibility. The Color-Word Interference Test is a Stroop test where, in condition 3, the participant has to inhibit an overlearned verbal response and name the dissonant colors of the words. In condition 4, the participant is asked to switch back and forth between naming the words and the dissonant colors. We also used completion time in seconds on the Trail Making Test, condition 4 (TMT4; Delis et al., 2001) as a measure of cognitive flexibility. In this test, the participant is asked to draw a line interchangeable between numbers and letters in the right alphabetical and sequential order. The Color-Word Interference Test and the TMT are widely used and have demonstrated good psychometric properties in a general population sample and individuals with depression (mean reliability = 0.62 to 0.90) (Delis et al., 2001; Homack et al., 2005; Wagner et al., 2011).

The four EF measures were administered at all time points. In line with evidence suggesting a unidimensional model of EFs in children and adolescents (Karr et al., 2018), and to reduce the number of variables and increase statistical and predictive power, we calculated a global EF composite score comprising all four measures (Bloemen et al., 2018; Øie et al., 2021; Owens & Hinshaw, 2016; Romer & Pizzagalli, 2021; Suchy & Brothers, 2022). This EF composite was supported by factor analysis showing high factor loadings (≥. 58). Test scores were converted to Z scores based on the mean and standard deviation of the TD group, in such a way that the new variables correlated perfectly with the original variables and retained all interindividual variance. The composite score was calculated so that higher scores indicate EF difficulties.

### Measure of ADHD symptoms

We used total scores on the parent-rated ADHD Rating Scale-IV (ARS-IV; DuPaul, Power, Anastoupolous, et al., 1998) to measure ADHD symptoms. This measure was only administered at T1 and T2. The ARS-IV has good reliability (α ≥ 0.80), test-retest reliability (*r* ≥ .78), factor structure, convergent and discriminant reliability on psychometric evaluation in general-population and clinically-referred samples (DuPaul, Power, McGoey, et al., 1998; Zhang et al., 2005). As expected, there was a high correlation between ADHD symptoms and the ADHD diagnosis in our sample (point biserial correlation *r* = .80, *p* < .001). ADHD symptoms were chosen because a dimensional variable preserves more variation between individuals and increases statistical power (Agresti & Finlay, 2014).

## Statistical analyses

Statistical analyses were conducted with SPSS version 26. Predictors of psychopathology symptoms were analyzed with hierarchical multiple regression analyses with psychopathology symptoms at T1, T2, and T3 as the dependent variables. The predictors were entered into the models in four steps. In step 1, sex and age were entered to serve as covariates in subsequent steps. In step 2, the T1 EFs composite score was added to assess the unique and independent predictive value of EFs. In step 3, T1 parent-rating of ADHD symptoms was added to assess the combined predictive value of EFs and ADHD symptoms. In step 4, an interaction term between EFs and sex was added to explore possible sex moderation. We assessed the increase in explained variance (*R*^2^) for each step. Lastly, we performed three regression analyses with EFs at T1, T2, and T3 as the dependent variables. Sex, age, T1 total psychopathology symptoms, and T1 ADHD symptoms were entered as independent variables to explore whether psychopathology symptoms predicted later EFs.

## Results

### Predictors of total psychopathology symptoms

**Fig. 1:**
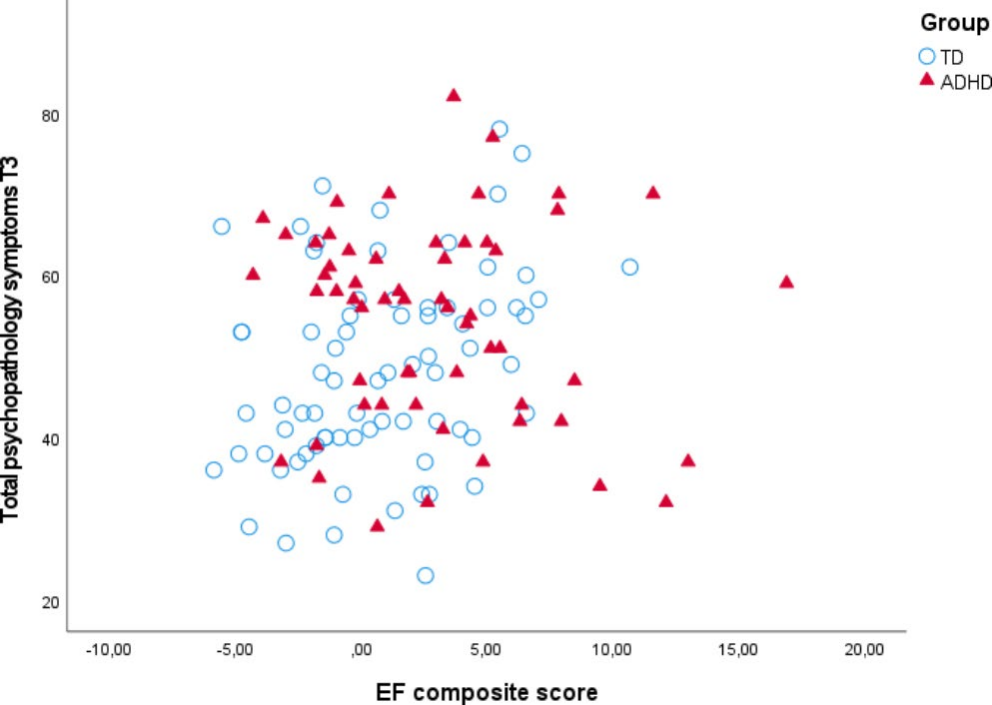
Scatter plot of the bivariate relationship between baseline executive function composite and T3 total psychopathology symptoms. (Note. TD = typically developing, ADHD = Attention-Deficit/Hyperactivity Disorder, T3 = 10-year follow-up, EF = Executive function)

Results from the three hierarchical regression analyses are presented in Table 4. At baseline, EFs explained 14% of the variance in total psychopathology symptoms beyond age and sex (Δ*R*^*2*^ = 0.14, Δ*F* = 20.03, *p* < .001). However, the effect of EFs was no longer significant when ADHD symptoms were added to the model, which explained an additional 57% of the variance in total psychopathology symptoms (Δ*R*^*2*^ = 0.57, Δ*F* = 256.55, *p* < .001). There was no evidence for a moderating role of sex (Δ*R*^*2*^ < 0.01, Δ*F* = 0.38, *p* = .538). At 2-year follow-up (T2), baseline EFs significantly predicted and explained 17% of the variance in T2 total psychopathology symptoms (Δ*R*^*2*^ = 0.17, Δ*F* = 24.67, *p* < .001). This effect remained significant (*p* = .017) after adding baseline ADHD symptoms, which accounted for an additional 38% of the variance (Δ*R*^*2*^ = 0.38, Δ*F* = 103.02, *p* < .001). There was no evidence for a moderating role of sex (Δ*R*^*2*^ < 0.01, Δ*F* = 0.09, *p* = .766). At 10-year follow-up (T3), baseline EFs continued to significantly predict and explain 12% of the variance in T3 total psychopathology symptoms (Δ*R*^*2*^ = 0.12, Δ*F* = 13.44, *p* < .001, Fig. 1). This effect remained significant (*p* = .009) after adding ADHD symptoms, which accounted for an additional 9% of the variance (Δ*R*^*2*^ = 0.09, Δ*F* = 11.42, *p* = .001). There was no evidence for a moderating role of sex (Δ*R*^*2*^ = 0.02, Δ*F* = 2.75, *p* = .100).

**Table 4:** Baseline predictors of total psychopathology symptoms at baseline, 2-year follow-up, and 10-year follow-up

		Baseline (T1)				2-year follow-up (T2)				10-year follow-up (T3)		
	Predictors (T1)	*B*	95% CI	*SE*		*B*	95% CI	*SE*		*B*	95% CI	*SE*
Step 1												
	Sex	3.03	[-2.08, 8.13]	2.58		1.11	[-3.93, 6.15]	2.55		3.24	[-1.89, 8.38]	2.59
	Age	− 0.48	[-1.75, 0.78]	0.64		− 0.37	[-1.61, 0.87]	0.63		0.84	[-0.42, 2.09]	0.63
Step 2												
	Sex	3.38	[-1.38, 8.14]	2.41		1.44	[-3.17, 6.06]	2.33		3.03	[-1.96, 7.84]	2.47
	Age	1.14	[-0.24, 2.52]	0.70		1.39	[0.06, 2.73]	0.67		2.19**	[0.80, 3.58]	0.70
	EF composite	1.38***	[0.77, 1.99]	0.31		1.47***	[0.89, 2.06]	0.30		1.24***	[0.57, 1.92]	0.34
Step 3												
	Sex	1.93	[-0.82, 4.67]	1.39		0.43	[-2.99, 3.84]	1.72		3.07	[-1.52, 7.65]	2.31
	Age	0.15	[-0.65, 0.96]	0.41		0.59	[-0.41, 1.58]	0.50		1.85**	[0.51, 3.18]	0.67
	EF composite	0.23	[-0.15, 0.61]	0.07		0.57*	[0.10, 1.04]	0.24		0.90**	[0.23, 1.57]	0.34
	ADHD symptoms	0.84***	[0.74, 0.95]	0.05		0.66***	[0.53, 0.79]	0.07		0.28**	[0.12, 0.45]	0.08
Step 4												
	Sex	2.33	[-0.71, 5.38]	1.54		0.18	[-3.62, 3.98]	1.92		1.60	[-3.27, 6.47]	2.45
	Age	0.14	[-0.67, 0.94]	0.41		0.60	[-0.41, 1.60]	0.51		1.94**	[0.62, 3.27]	0.67
	EF composite	0.32	[-0.16, 0.80]	0.24		0.52	[-0.07, 1.11]	0.30		0.43	[-0.44, 1.30]	0.44
	ADHD symptoms	0.84***	[0.74, 95]	0.05		0.66***	[0.53, 0.79]	0.07		0.30**	[0.13, 0.46]	0.08
	EF composite x Sex	− 0.19	[-0.79, 0.42]	0.31		0.11	[-0.63, 0.85]	0.38		0.91	[-0.180, 2.00]	0.55

*Note.* Dependent variable = total problems, Achenbach System of Empirically Based Assessment; *p < .05, ** p < .01, *** p < .001. EF = executive function, ADHD = attention-deficit/hyperactivity disorder

To explore whether the effect of EFs on psychopathology symptoms is limited to specific tests, we conducted follow-up regression analyses where total psychopathology symptoms at T1, T2, and T3 were the dependent variables, and sex, age, and the four tests of EFs were the independent variables. The results are shown in Table 5. Better working memory had a significant negative effect on total psychopathology symptoms concurrently at baseline (*p* = .013). Poorer working memory (*p* = .044) and cognitive flexibility on the TMT4 (*p* = .002) at baseline predicted more total psychopathology symptoms at T2. Poorer cognitive flexibility on the CW4 (*p* = .019) and the TMT4 (*p* = .040) at baseline predicted more total psychopathology symptoms at T3.

**Table 5:** Baseline executive functions as predictors of total psychopathology symptoms at baseline, 2-year follow-up, and 10-year follow-up

	Baseline (T1)				2-year follow-up (T2)				10-year follow-up (T3)		
Predictors (T1)	*B*	95% CI	*SE*		*B*	95% CI	*SE*		*B*	95% CI	*SE*
Sex	3.65	[-1.16, 8.45]	2.43		1.68	[-2.92, 6.27]	2.32		3.14	[-1.75, 8.03]	2.46
Age	1.05	[-0.35, 2.45]	0.71		1.35*	[0.01, 2.60]	0.68		1.95**	[0.53, 3.37]	0.71
LNS	-1.26*	[-2.25, − 0.27]	0.50		− 0.96*	[-1.90, − 0.03]	0.47		− 0.55	[-1.72, 0.63]	0.59
CW3	0.05	[-0.10, 0.20]	0.07		0.01	[-0.13, 0.15]	0.07		− 0.11	[-0.27, 0.05]	0.08
CW4	0.01	[-0.13, 0.16]	0.07		0.03	[-0.11, 0.17]	0.07		0.19*	[0.03, 0.35]	0.08
TMT4	0.04	[-0.02, 0.11]	0.03		0.10**	[0.04, 0.16]	0.03		0.07**	[0.00, 0.13]	0.03

*Note.* Dependent variable = total problems, Achenbach System of Empirically Based Assessment; *p < .05, ** p < .01, *** p < .001. EF = executive function, ADHD = attention-deficit/hyperactivity disorder, LNS = Letter-Number Sequencing, CW3 = Color-Word Interference Test, condition 3, CW4 = Color-Word Interference Test, condition 4, TMT4 = Trail Making Test, condition 4

## Predictors of internalizing symptoms

Results from the three hierarchical regression analyses are presented in Table S2. At baseline, EFs explained 8% of the variance in internalizing symptoms beyond age and sex (Δ*R*^*2*^ = 0.08, Δ*F* = 11.04, *p* = .001). However, the effect of EFs was no longer significant when ADHD symptoms were added to the model, which explained an additional 35% of the variance in internalizing symptoms (Δ*R*^*2*^ = 0.35, Δ*F* = 78.04, *p* < .001). There was no evidence for a moderating role of sex (Δ*R*^*2*^ < 0.01, Δ*F* = 0.41, *p* = .523). At 2-year follow-up (T2), baseline EFs significantly predicted and explained 13% of the variance in T2 internalizing symptoms (Δ*R*^*2*^ = 0.13, Δ*F* = 17.52, *p* < .001). This effect remained significant (*p* = .032) after adding baseline ADHD symptoms, which accounted for an additional 19% of the variance (Δ*R*^*2*^ = 0.193, Δ*F* = 34.242, *p* < .001). There was no evidence for a moderating role of sex (Δ*R*^*2*^ < 0.01, Δ*F* = 0.01, *p* = .946). At 10-year follow-up (T3), baseline EFs continued to significantly predict and explain 10% of the variance in T3 internalizing symptoms (Δ*R*^*2*^ = 0.10, Δ*F* = 10.15, *p* = .002). This effect remained significant (*p* = .015) after adding ADHD symptoms, which accounted for an additional 4% of the variance (Δ*R*^*2*^ = 0.04, Δ*F* = 3.98, *p* = .049). There was no evidence for a moderating role of sex (Δ*R*^*2*^ = 0.02, Δ*F* = 2.54, *p* = .115).

To explore whether the effect of EFs on internalizing symptoms is limited to specific tests, we conducted follow-up regression analyses where internalizing symptoms at T1, T2, and T3 were the dependent variables, and sex, age, and the four tests of EFs were the independent variables. The results are shown in Table S3. At baseline, no EFs were concurrently associated with internalizing symptoms. Poorer cognitive flexibility on TMT4 at baseline predicted more internalizing symptoms at T2 (*p* < .001). Poorer response inhibition at baseline predicted fewer internalizing symptoms at T3 (*p* = .026). Poorer cognitive flexibility on the CW4 (*p* = .022) and TMT4 (*p* = .031) at baseline predicted more internalizing symptoms at T3.

## Predictors of externalizing symptoms

Results from the three hierarchical regression analyses are presented in Table S4. At baseline, EFs explained 11% of the variance in externalizing symptoms beyond age and sex (Δ*R*^*2*^ = 0.11, Δ*F* = 15.26, *p* < .001). However, the effect of EFs was no longer significant when ADHD symptoms were added to the model, which explained an additional 47% of the variance in externalizing symptoms (Δ*R*^*2*^ = 0.47, Δ*F* = 138.76, *p* < .001). There was no evidence for a moderating role of sex (Δ*R*^*2*^ = 0.01, Δ*F* = 1.57, *p* = .212). At 2-year follow-up (T2), baseline EFs significantly predicted and explained 14% of the variance in T2 externalizing symptoms (Δ*R*^*2*^ = 0.14, Δ*F* = 19.32, *p* < .001). This effect remained significant (*p* = .049) after adding baseline ADHD symptoms, which accounted for an additional 30% of the variance (Δ*R*^*2*^ = 0.30, Δ*F* = 65.48, *p* < .001). There was no evidence for a moderating role of sex (Δ*R*^*2*^ < 0.01, Δ*F* < 0.01, *p* = .959). At 10-year follow-up (T3), baseline EFs continued to significantly predict and explain 11% of the variance in T3 externalizing symptoms (Δ*R*^*2*^ = 0.11, Δ*F* = 12.13, *p* = .001). This effect remained significant (*p* = .014) after adding ADHD symptoms, which accounted for an additional 9% of the variance (Δ*R*^*2*^ = 0.09, Δ*F* = 10.96, *p* = .001). There was no evidence for a moderating role of sex (Δ*R*^*2*^ = 0.01, Δ*F* = 1.23, *p* = .270).

To explore whether the effect of EFs on psychopathology symptoms is limited to specific tests, we conducted follow-up regression analyses where externalizing symptoms at T1, T2, and T3 were the dependent variables, and sex, age, and the four tests of EFs were the independent variables. The results are shown in Table S5. Better working memory had a significant negative effect on externalizing symptoms concurrently at baseline (*p* = .005). Poorer cognitive flexibility on the TMT4 at baseline predicted more externalizing symptoms at T2 (*p* = .017). Poorer cognitive flexibility on the CW4 (*p* = .020) at baseline predicted more externalizing symptoms at T3.

## Childhood psychopathology symptoms as a predictor of executive functions

There was no evidence of an effect of childhood psychopathology symptoms on concurrent or later EFs (see Table 6).

**Table 6:** Baseline predictors of executive function composite score at baseline, 2-year follow-up, and 10-year follow-up

	Baseline (T1)				2-year follow-up (T2)				10-year follow-up (T3)		
Predictors (T1)	*B*	95% CI	*SE*		*B*	95% CI	*SE*		*B*	95% CI	*SE*
Sex	− 0.49	[-1.77, 0.80]	0.65		0.07	[-1.23, 1.38]	0.66		0.24	[-0.98, 1.45]	0.61
Age	-1.13***	[-1.44, − 0.81]	0.16		− 0.82***	[-1.14, − 0.50]	0.16		− 0.15	[-0.45, 0.15]	0.15
CBCL total problems	0.05	[-0.03, 0.13]	0.04		0.05	[-0.03, 0.13]	0.04		0.07	[-0.00, 0.15]	0.04
ADHD symptoms	0.06	[-0.02, 0.15]	0.04		0.03	[-0.06, 0.11]	0.04		0.01	[-0.07, 0.09]	0.04

*Note.* Dependent variable = Executive function composite score; *p < .05, ** p < .01, *** p < .001. CBCL = Child Behavior Checklist, ADHD = attention-deficit/hyperactivity disorder

## Discussion

Our study provides the field with at least four pieces of information. First, we replicated and extended previous findings showing that EFs predicted later psychopathology symptoms in a sample of both boys and girls with ADHD and with a different set of neuropsychological tests, suggesting that previous results can be replicated and generalized (Meza et al., 2020; Miller et al., 2012; Owens & Hinshaw, 2016; Rinsky & Hinshaw, 2011). Second, we found that EFs remained a significant longitudinal predictor in conjunction with ADHD symptoms. Third, we found that the effect of EFs on psychopathology symptoms at the 10-year follow-up was accounted for by cognitive flexibility. Fourth, we found no effect of childhood psychopathology symptoms on later EFs, so our study suggests a directional relationship from childhood impairments in EFs to later psychopathology symptoms. Thus, our findings underscore the long-term prospective longitudinal importance of EFs and their impact on psychopathology symptoms in emerging adulthood.

When examining specific relations between EFs and psychopathology symptoms, our results suggest that the impact of EFs is quite similar across total psychopathology, internalizing, and externalizing symptoms. This is in line with other findings suggesting that impairments in EFs are a transdiagnostic risk factor for psychopathology (Bloemen et al., 2018; Romer & Pizzagalli, 2021; Zelazo, 2020). In line with evidence suggesting a unidimensional model of EFs in childhood and adolescence (Karr et al., 2018), and evidence suggesting that such an EF composite has greater predictive value (Bloemen et al., 2018; Romer & Pizzagalli, 2021), we focused on the EF composite score in this study. This EF composite score explained a similar amount of variance in total psychopathology, internalizing, and externalizing symptoms at the 10-year follow-up (12, 10, and 11%, respectively). However, exploratory analyses suggested that the longitudinal effects of EFs on later psychopathology symptoms were accounted for by cognitive flexibility (at the 10-year follow-up). It is difficult from our data to determine whether the effects of EFs are limited to specific domains such as cognitive flexibility or by a general and broad impairment in EFs. The finding that cognitive flexibility may be especially important for later psychopathology symptoms is in line with a recent finding among individuals with autism spectrum disorder (Hollocks et al., 2021). Similarly, it has been proposed that cognitive inflexibility is a transdiagnostic risk factor of psychopathology as it contributes to a lack of goal attainment (causing anger and frustration) and rumination and worrying (causing depression and anxiety) (Morris & Mansell, 2018). The relationship between cognitive inflexibility and psychopathology has particularly been noted for internalizing symptoms (Bloemen et al., 2018; Yang et al., 2017), and in line with this, both tests of cognitive flexibility (CW4 and TMT4) predicted later internalizing symptoms whereas only the CW4 predicted later externalizing symptoms.

ADHD symptoms were significantly associated with psychopathology symptoms across all time points. EFs were not a significant correlate cross-sectionally (after adding ADHD symptoms to the analysis), but EFs were a significant longitudinal predictor at 2- and 10-year follow-up. The unique variance explained by baseline EFs was relatively stable over time (14, 17, & 12% of the variance in total psychopathology symptoms) whereas the additional variance explained by baseline ADHD symptoms declined markedly over time (from 57 to 9%). One possible explanation of this decline in explained variance is that associations between baseline ADHD symptoms and psychopathology symptoms at baseline and T2 were subject to a within-informant effect (i.e., all measures were based on parent ratings), whereas the association between baseline ADHD symptoms and psychopathology symptoms at 10-year follow-up reflects a cross-informant effect (i.e., from parent-rating to self-report). It is well-known that the agreement between children and their parents in ratings of the same construct is often moderate at best (van der Ende et al., 2012), and that within-informant associations tend to be inflated (DeYoung, 2006; Martel, 2009). Thus, associations across different informants or measures (e.g., rating scales and neuropsychological tests) tend to be more valid (Owens & Hinshaw, 2016). Consequently, the most interesting findings from this study are the effect of baseline EFs, measured by neuropsychological tests, and baseline ADHD symptoms, measured by parent ratings, on self-reported psychopathology symptoms at the 10-year follow-up. We speculate on whether the high and probably inflated association between ADHD symptoms and psychopathology symptoms concurrently may obscure an important effect of EFs. However, Romer & Pizzagalli (2021) argued based on their findings that the effect of impaired EFs on later psychopathology symptoms may be more pronounced over longer than shorter periods of time, an interpretation which is in line with the findings presented herein as well. It may be easier to detect the effect of EFs in the long run because, over time, EFs may influence psychopathology symptoms through several different pathways (e.g., self-regulation, academic achievement, occupational functioning, social skills) (Owens & Hinshaw, 2016). Therefore, the true effect of EFs may be difficult to establish in cross-sectional designs, and thus, longitudinal studies are needed.

Further research is needed on the pathways from impaired EFs to later psychopathology, but some mechanisms can be hypothesized. First, EFs subserve emotion regulation by allowing an individual to inhibit prepotent responses arising from emotional arousal (i.e., response inhibition), to hold response choices and long-term goals in mind to guide behavior (i.e., working memory), to flexibly adapt to situational demands (i.e., cognitive flexibility), and to take different perspectives on the situation (i.e., working memory and cognitive flexibility) (Diamond, 2013; Zelazo & Cunningham, 2007). Consequently, previous studies have discussed whether the predictive value of EFs on later psychopathology symptoms may be mediated by emotion regulation (e.g., Miller et al., 2012; Rinsky & Hinshaw, 2011; Strugstad et al., 2018). Second, EFs influence outcomes in important areas such as academic achievements and occupational functioning (Miller et al., 2012; Owens & Hinshaw, 2016). School failure and poor academic achievements are difficult starting points for emerging adults who are expected to either study or begin their career and be financially independent. Impaired EFs may make it difficult to plan and organize work tasks, show up on time, and focus on work tasks in the face of distractions. Thus, emerging adults with executive dysfunction may fail to meet society’s expectations in this phase of life, enhancing the risk for psychopathology (Schwartz & Petrova, 2019).

## Limitations

A few limitations of our study should be noted. First, the sample size was relatively modest. Together with some attrition over time, this may limit the generalizability of our findings. Second, the age range of our sample was relatively wide (8 to 17 years at baseline) and may represent a limitation, but standard deviations (around 2 years) suggest that most participants were around the mean age. Third, our sample of participants with ADHD was restricted to clinic-referred children which may differ from the general ADHD population.

## Conclusions

Our findings show that childhood EFs predict later psychopathology symptoms, even beyond the effect of ADHD symptoms. Targeting EFs in interventions for children and adolescents with ADHD may therefore be important to prevent psychopathology symptoms and improve adult outcomes. It may be the combined effect of EFs that is predictive of later psychopathology outcomes (Owens & Hinshaw, 2016) and our results suggest that there may be different EFs affecting psychopathology in the short and long run. Thus, it may be necessary to target multiple domains of EFs (i.e., inhibition, cognitive flexibility, working memory) instead of focusing on a single neuropsychological deficit (Cortese et al., 2015). The effects of EF interventions may be small in the short-run (Lambez et al., 2020) but still have important influences on long-term outcomes (Romer & Pizzagalli, 2021).

## Electronic supplementary material

Below is the link to the electronic supplementary material.

Supplementary Material 1
