# Supplementary material for: Childhood executive functions and ADHD symptoms predict psychopathology symptoms in emerging adults with and without ADHD: a 10-year longitudinal study
Source: Res Child Adolesc Psychopathol. 2022 Oct 4;51(2):261–71. doi: 10.1007/s10802-022-00957-7 (PMC9867664; doi:10.1007/s10802-022-00957-7)
Supplement: Supplementary file 1 — Supplementary Material 1 [file 10802_2022_957_MOESM1_ESM.docx]

Table S1

*Zero-order correlations between study variables.*

|  | 1. | 2. | 3. | 4. | 5. | 6. | 7. | 8. | 9. | 10. | 11. | 12. | 13. | 14. | 15. | 16. | 17. | 18. | 19. | 20. | 21. | 22. | 23. | 24. | 25. | 26. | 27. |
| --- | --- | --- | --- | --- | --- | --- | --- | --- | --- | --- | --- | --- | --- | --- | --- | --- | --- | --- | --- | --- | --- | --- | --- | --- | --- | --- | --- |
| 1. Age | - |  |  |  |  |  |  |  |  |  |  |  |  |  |  |  |  |  |  |  |  |  |  |  |  |  |  |
| 2. T1 ASEBA total | -.07 | - |  |  |  |  |  |  |  |  |  |  |  |  |  |  |  |  |  |  |  |  |  |  |  |  |  |
| 3. T1 ASEBA intern | -.05 | .84** | - |  |  |  |  |  |  |  |  |  |  |  |  |  |  |  |  |  |  |  |  |  |  |  |  |
| 4. T1 ASEBA extern | .01 | .89** | .63** | - |  |  |  |  |  |  |  |  |  |  |  |  |  |  |  |  |  |  |  |  |  |  |  |
| 5. T1 LNS | -.39** | .35** | .22* | .30** | - |  |  |  |  |  |  |  |  |  |  |  |  |  |  |  |  |  |  |  |  |  |  |
| 6. T1 CW3 | -.53** | .27** | .26** | .17 | .59** | - |  |  |  |  |  |  |  |  |  |  |  |  |  |  |  |  |  |  |  |  |  |
| 7. T1 CW4 | -.47** | .25** | .23** | .17 | .58** | .77** | - |  |  |  |  |  |  |  |  |  |  |  |  |  |  |  |  |  |  |  |  |
| 8. T1 TMT | -.42** | .26** | .23** | .18* | .50** | .53** | .45** | - |  |  |  |  |  |  |  |  |  |  |  |  |  |  |  |  |  |  |  |
| 9. T1 EF composite | -.54** | .34** | .27** | .26** | .83** | .87** | .85** | .74** | - |  |  |  |  |  |  |  |  |  |  |  |  |  |  |  |  |  |  |
| 10. T1 ARS-IV | -.05 | .85** | .66** | .75** | .36** | .23** | .24** | .26** | .34** | - |  |  |  |  |  |  |  |  |  |  |  |  |  |  |  |  |  |
| 11. T2 ASEBA total | -.07 | .81** | .75** | .68** | .35** | .26** | .27** | .38** | .37** | .74** | - |  |  |  |  |  |  |  |  |  |  |  |  |  |  |  |  |
| 12. T2 ASEBA intern | .02 | .64** | .73** | .48** | .23** | .19* | .24** | .36** | .29** | .55** | .89** | - |  |  |  |  |  |  |  |  |  |  |  |  |  |  |  |
| 13. T2 ASEBA extern | .01 | .76** | .63** | .76** | .28** | .20* | .20* | .28** | .29** | .66** | .88** | .70** | - |  |  |  |  |  |  |  |  |  |  |  |  |  |  |
| 14. T2 LNS | -.35** | .33** | .22* | .29** | .68** | .66** | .60** | .50** | .76** | .25** | .38** | .23** | .36** | - |  |  |  |  |  |  |  |  |  |  |  |  |  |
| 15. T2 CW3 | -.46** | .22* | .18* | .16 | .55** | .78** | .69** | .58** | .77** | .22* | .30** | .20* | .23** | .67** | - |  |  |  |  |  |  |  |  |  |  |  |  |
| 16. T2 CW4 | -.47** | .22* | .14 | .12 | .52** | .72** | .74** | .56** | .77** | .22* | .28** | .21* | .18* | .62** | .83** | - |  |  |  |  |  |  |  |  |  |  |  |
| 17. T2 TMT | -.33** | .25* | .17 | .18* | .55** | .53** | .45** | .59** | .64** | .23* | .30** | .15 | .27** | .71** | .61** | .53** | - |  |  |  |  |  |  |  |  |  |  |
| 18. T2 EF composite | -.46** | .30* | .20* | .23* | .67** | .81** | .75** | .63** | .86** | .26** | .37** | .22** | .31** | .89** | .89** | .85** | .82** | - |  |  |  |  |  |  |  |  |  |
| 19. T2 ARS-IV | -.09 | .67** | .60** | .58** | .22* | .23** | .19* | .22* | .26** | .71** | .79** | .68** | .69** | .31** | .23* | .23** | .17 | .28** | - |  |  |  |  |  |  |  |  |
| 20. T3 ASEBA total | .15 | .38** | .35** | .29** | .15 | .03 | .19 | .18 | .17 | .38** | .42** | .36** | .31** | .17 | .13 | .08 | .11 | .16 | .28** | - |  |  |  |  |  |  |  |
| 21. T3 ASEBA intern | .11 | .27** | .28** | .16 | .18 | -.01 | .16 | .19 | .16 | .26* | .37** | .34** | .24* | .14 | .09 | .06 | .10 | .13 | .20* | .90** | - |  |  |  |  |  |  |
| 22. T3 ASEBA extern | .17 | .36** | .29** | .32** | .09 | .09 | .22* | .09 | .15 | .37** | .30** | .22* | .25* | .17 | .16 | .10 | .07 | .17 | .27** | .84** | .61** | - |  |  |  |  |  |
| 23. T3 LNS | -.05 | .37** | .27** | .37** | .63** | .48** | .49** | .37** | .60** | .30** | .37** | .26** | .38** | .74** | .49** | .45** | .52** | .66** | .28** | .28** | .23* | .27** | - |  |  |  |  |
| 24. T3 CW3 | -.23* | .32** | .28** | .29** | .66** | .74** | .66** | .44** | .76** | .29** | .30** | .20* | .29** | .74** | .79** | .64** | .61** | .80** | .24* | .14 | .08 | .20 | .56** | - |  |  |  |
| 25. T3 CW4 | -.19 | .36** | .36** | .29** | .53** | .62** | .61** | .36** | .65** | .28** | .36** | .32** | .31** | .63** | .65** | .71** | .43** | .69** | .31** | .16 | .09 | .23* | .47** | .70** | - |  |  |
| 26. T3 TMT | -.04 | .16 | .17 | .13 | .50** | .41** | .30** | .44** | .51** | .17 | .25* | .22* | .25* | .45** | .41** | .38** | .56** | .51** | .25* | .06 | .04 | .04 | .40** | .50** | .43** | - |  |
| 27. T3 EF composite | -.13 | .39** | .33* | .36** | .74** | .67** | .63** | .49** | .78** | .34** | .42** | .32** | .40** | .82** | .69** | .64** | .64** | .82** | .34** | .23* | .17 | .25* | .87** | .81** | .75** | .69** | - |

*Note.* ASEBA = Achenbach System of Empirically Based Assessment, LNS = Letter-number sequencing, CW = Color-Word test, TMT = Trail Making Test, EF = Executive Function, ARS-IV = ADHD Rating Scale IV edition, **p* ≤ .05, ***p* ≤ .01.

Table S2.

*Baseline predictors of internalizing symptoms at baseline, two-year follow-up, and 10-year follow-up.*

|  |  | Baseline (T1) | | |  | Two-year follow-up (T2) | | |  | 10-year follow-up (T3) | | |
| --- | --- | --- | --- | --- | --- | --- | --- | --- | --- | --- | --- | --- |
|  | Predictors (T1) | *B* | 95% CI | *SE* |  | *B* | 95% CI | *SE* |  | *B* | 95% CI | *SE* |
| Step 1 | |  |  |  |  | | |  |  | | |  |
|  | Sex | 1.56 | [-2.98, 6.10] | 2.29 |  | .58 | [-4.07, 5.21] | 2.34 |  | 3.94 | [-1.06, 8.93] | 2.52 |
|  | Age | -.28 | [-1.40, .85] | .57 |  | .19 | [-.95, 1.32] | .58 |  | .51 | [-.71, 1.73] | .62 |
| Step 2 | |  |  |  |  | | |  |  | | |  |
|  | Sex | 1.80 | [-2.58, 6.17] | 2.21 |  | .84 | [-3.52, 5.20] | 1.96 |  | 3.75 | [-1.02, 8.53] | 2.40 |
|  | Age | .83 | [-.44, 2.10] | .64 |  | 1.59 | [.33, 2.85] | .64 |  | 1.68* | [.31, 3.05] | .69 |
|  | EF composite | .94** | [.38, 1.50] | .28 |  | 1.17*** | [.62, 1.73] | .28 |  | 1.07** | [.40, 1.73] | .34 |
| Step 3 | |  |  |  |  | | |  |  | | |  |
|  | Sex | .79 | [-2.66, 4.24] | 1.74 |  | .17 | [-3.70, 4.04] | 1.96 |  | 3.77 | [-.92, 8.47] | 2.37 |
|  | Age | .14 | [-.87, 1.15] | .51 |  | 1.06 | [-.07, 2.19] | .57 |  | 1.47* | [.10, 2.84] | .69 |
|  | EF composite | .14 | [-.34, .62] | .24 |  | .58* | [.05, 1.11] | .27 |  | .86* | [.17, 1.55] | .35 |
|  | ADHD symptoms | .59*** | [.46, .72] | .07 |  | .43*** | [.28, .57] | .07 |  | .17* | [.00, .34] | .09 |
| Step 4 | |  |  |  |  |  |  |  |  |  |  |  |
|  | Sex | 1.32 | [-2.51, 5.15] | 1.94 |  | .11 | [-4.20, 4.42] | 2.18 |  | 2.33 | [-2.67, 7.33] | 2.52 |
|  | Age | .12 | [-.90, 1.14] | .51 |  | 1.06 | [-.08, 2.20] | .57 |  | 1.57* | [.20, 2.93] | .69 |
|  | EF composite | .26 | [-.34, 86] | .30 |  | .57 | [-.10, 1.24] | .34 |  | .39 | [-.50, 1.29] | .45 |
|  | ADHD symptoms | .58*** | [.45, 72] | .07 |  | .43*** | [.28, .58] | .07 |  | .19* | [.02, .36] | .09 |
|  | EF composite x Sex | -.246 | [-1.01, .52] | .38 |  | .03 | [-.81, .87] | .43 |  | .90 | [-.22, 2.02] | .56 |

*Note.* Dependent variable = total problems, Achenbach System of Empirically Based Assessment; **p < 0.05, ** p < 0.01, *** p < 0.001*. EF = executive function, ADHD = attention-deficit/hyperactivity disorder.

Table S3.

*Baseline executive functions as predictors of internalizing symptoms at baseline, two-year follow-up, and 10-year follow-up.*

|  |  | Baseline (T1) | | |  | Two-year follow-up (T2) | | |  | 10-year follow-up (T3) | | |
| --- | --- | --- | --- | --- | --- | --- | --- | --- | --- | --- | --- | --- |
|  | Predictors (T1) | *B* | 95% CI | *SE* |  | *B* | 95% CI | *SE* |  | *B* | 95% CI | *SE* |
|  | Sex | 1.94 | [-2.50, 6.38] | 2.24 |  | .75 | [-3.53, 5.03] | 2.16 |  | 3.56 | [-1.17, 8.30] | 2.38 |
|  | Age | .90 | [-.390, 2.20] | .65 |  | 1.63* | [.38, 2.88] | .63 |  | 1.32 | [-.05, 2.70] | .69 |
|  | LNS | -.28 | [-1.20, .63] | .46 |  | -.23 | [-1.10, .64] | .44 |  | -.91 | [-2.04, .23] | .57 |
|  | CW3 | .08 | [-.05, .22] | .07 |  | -.03 | [-.16, .10] | .06 |  | -.17* | [-.32, -.02] | .08 |
|  | CW4 | .02 | [-.12, .15] | .07 |  | .08 | [-.05, .21] | .07 |  | .18* | [.03, .33] | .08 |
|  | TMT4 | .04 | [-.02, .10] | .03 |  | .11*** | [.06, .17] | .03 |  | .07* | [.01, .13] | .03 |

*Note.* Dependent variable = total problems, Achenbach System of Empirically Based Assessment; **p < 0.05, ** p < 0.01, *** p < 0.001*. EF = executive function, ADHD = attention-deficit/hyperactivity disorder.

Table S4.

*Baseline predictors of externalizing symptoms at baseline, two-year follow-up, and 10-year follow-up.*

|  |  | Baseline (T1) | | |  | Two-year follow-up (T2) | | |  | 10-year follow-up (T3) | | |
| --- | --- | --- | --- | --- | --- | --- | --- | --- | --- | --- | --- | --- |
|  | Predictors (T1) | *B* | 95% CI | *SE* |  | *B* | 95% CI | *SE* |  | *B* | 95% CI | *SE* |
| Step 1 | |  |  |  |  | | |  |  | | |  |
|  | Sex | 1.41 | [-3.29, 6.12] | 2.38 |  | 1.72 | [-2.49, 5.93] | 2.13 |  | 1.42 | [-3.15, 5.99] | 2.30 |
|  | Age | .17 | [-1.00, 1.33] | .59 |  | .11 | [-.93, 1.14] | .52 |  | .89 | [-.22, 2.01] | .56 |
| Step 2 | |  |  |  |  | | |  |  | | |  |
|  | Sex | 1.70 | [-2.76, 6.16] | 2.25 |  | 1.97 | [-1.96, 5.90] | 1.98 |  | 1.24 | [-3.08, 5.56] | 2.18 |
|  | Age | 1.49* | [.20, 2.79] | .65 |  | 1.43* | [.30, 2.57] | .57 |  | 2.05** | [.80, 3.29] | .63 |
|  | EF composite | 1.13*** | [.56, 1.70] | .29 |  | 1.11*** | [.61, 1.61] | .25 |  | 1.06** | [.45, 1.66] | .30 |
| Step 3 | |  |  |  |  | | |  |  | | |  |
|  | Sex | .50 | [-2.59, 3.59] | 1.56 |  | 1.21 | [-1.97, 4.39] | 1.61 |  | 1.27 | [-2.84, 5.38] | 2.07 |
|  | Age | .68 | [-.23, 1.58] | .46 |  | .83 | [-.10, 1.76] | .47 |  | 1.74** | [.55, 2.94] | .60 |
|  | EF composite | .17 | [-.25, .60] | .22 |  | .44* | [.00, .87] | .22 |  | .76* | [.16, 1.36] | .30 |
|  | ADHD symptoms | .70*** | [.58, .82] | .06 |  | .49*** | [.37, .61] | .06 |  | .25** | [.10, .40] | .08 |
| Step 4 | |  |  |  |  |  |  |  |  |  |  |  |
|  | Sex | 1.42 | [-1.99, 4.84] | 1.72 |  | 1.25 | [-2.29, 4.79] | 1.79 |  | .38 | [-4.02, 4.78] | 2.22 |
|  | Age | .63 | [-.27, 1.54] | .46 |  | .83 | [-.10, 1.77] | .47 |  | 1.80** | [.60, 3.00] | .60 |
|  | EF composite | .38 | [-.16, .92] | .27 |  | .45 | [-.10, 1.00] | .28 |  | .47 | [-.32, 1.26] | .40 |
|  | ADHD symptoms | .70*** | [.58, .81] | .06 |  | .49*** | [.37, .61] | .06 |  | .26** | [.11, .41] | .08 |
|  | EF composite x Sex | -.43 | [-1.11, .25] | .34 |  | -.02 | [-.71, .67] | .35 |  | .55 | [-.44, 1.53] | .50 |

*Note.* Dependent variable = total problems, Achenbach System of Empirically Based Assessment; **p < 0.05, ** p < 0.01, *** p < 0.001*. EF = executive function, ADHD = attention-deficit/hyperactivity disorder.

Table S5.

*Baseline executive functions as predictors of externalizing symptoms at baseline, two-year follow-up, and 10-year follow-up.*

|  |  | Baseline (T1) | | |  | Two-year follow-up (T2) | | |  | 10-year follow-up (T3) | | |
| --- | --- | --- | --- | --- | --- | --- | --- | --- | --- | --- | --- | --- |
|  | Predictors (T1) | *B* | 95% CI | *SE* |  | *B* | 95% CI | *SE* |  | *B* | 95% CI | *SE* |
|  | Sex | 1.93 | [-2.55, 6.41] | 2.26 |  | 2.19 | [-1.76, 6.14] | 2.00 |  | 1.63 | [-2.78, 6.03] | 2.22 |
|  | Age | 1.35* | [.04, 2.65] | .66 |  | 1.42* | [.26, 2.57] | .58 |  | 2.02** | [.74, 3.30] | .64 |
|  | LNS | -1.33** | [-2.26, -.41] | .47 |  | -.70 | [-1.51, .11] | .41 |  | .02 | [-1.04, 1.07] | .53 |
|  | CW3 | .02 | [-.12, .15] | .07 |  | .02 | [-.10, .14] | .06 |  | -.01 | [-.15, .14] | .07 |
|  | CW4 | .01 | [-.13, .14] | .07 |  | .02 | [-.10, .14] | .06 |  | .17* | [.03, .31] | .07 |
|  | TMT4 | .03 | [-.03, .09] | .03 |  | .07* | [.01, .12] | .03 |  | .03 | [-.03, .09] | .03 |

*Note.* Dependent variable = total problems, Achenbach System of Empirically Based Assessment; **p < 0.05, ** p < 0.01, *** p < 0.001*. EF = executive function, ADHD = attention-deficit/hyperactivity disorder.

**Figures**

Figure S1.

*Scatter plot of the bivariate relationship between baseline executive function composite and baseline total psychopathology symptoms.*


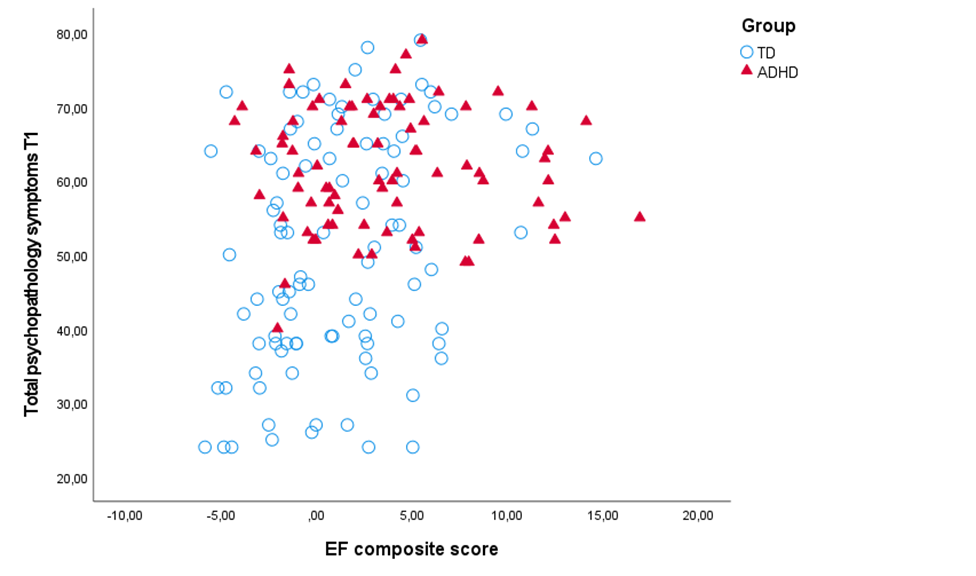


*Note.* TD = typically developing, ADHD = Attention-Deficit/Hyperactivity Disorder, T1 = baseline, EF = Executive function.

Figure S2.

*Scatter plot of the bivariate relationship between baseline executive function composite and T2 total psychopathology symptoms.*


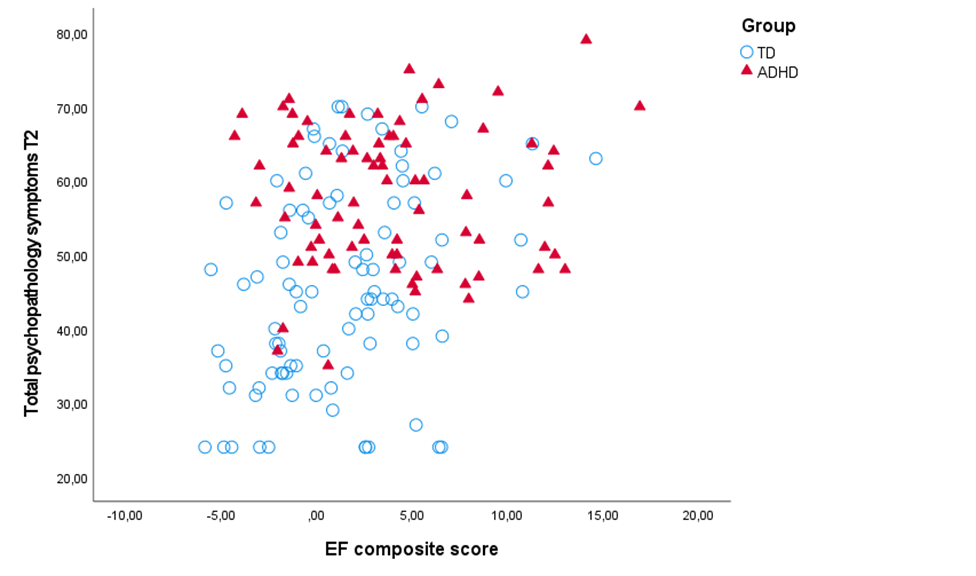


*Note.* TD = typically developing, ADHD = Attention-Deficit/Hyperactivity Disorder, T2 = two-year follow-up, EF = Executive function.
